# Correction to “5‐Aminolevulinic Acid Improves Immune Response in Dairy Cows In Vivo and In Vitro”

**DOI:** 10.1111/asj.70234

**Published:** 2026-08-19

Hendawy, A. O. , Sugimura, S. , Taniguchi, S. , Sato, K. 2026. “5‐Aminolevulinic Acid Improves Immune Response in Dairy Cows In Vivo and In Vitro.” Animal Science Journal, 97, no. 1: e70222. 10.1111/asj.70222.42530399 PMC13422138

Affiliation 4 was inadvertently omitted from the affiliations of the author Amin Omar Hendawy and has been added. In addition, Affiliation 4 was incorrectly assigned to the author Kan Sato and has been replaced with Affiliation 2.

We apologize for this error.

